# Introduction to the thematic review series on intracellular protein degradation. The ubiquitous biology of intracellular protein degradation: A tribute to Alfred L. (“Fred”) Goldberg

**DOI:** 10.1016/j.jbc.2026.113311

**Published:** 2026-07-01

**Authors:** George N. DeMartino

**Affiliations:** Department of Physiology, University of Texas Southwestern Medical Center, Dallas, Texas, USA

This JBC thematic review series on Intracellular Protein Degradation is dedicated to Alfred L. “Fred” Goldberg, who died in April 2023 at the age of 80. Fred was a pioneer, leader, and champion of the field. His foundational discoveries in the 1960s, his sustained advances over six subsequent decades, and his visionary leadership played major roles in the rise of protein degradation from a topic whose importance was initially ignored or dismissed to one now recognized to mediate or regulate nearly every aspect of cellular function. Fred’s practice of viewing biochemical and mechanistic data in a physiologic context helped establish the biologic significance of protein degradation and explained how the process was involved in human diseases. The articles in this thematic review series are selective snapshots of some of the many contemporary topics that resulted from or were inspired by Fred’s work.

## Covalent modification of proteins by ubiquitin and ubiquitin-like proteins

Fred’s discovery of ATP-dependent protein degradation in cell-free extracts of reticulocytes was the foundation for the identification and initial dissection of protein ubiquitylation by Hershko, Ciechanover, and Rose (1, 2). Ubiquitylation and analogous covalent modifications by subsequently identified ubiquitin-like proteins are now known to play pervasive roles in biology. For example, ubiquitylation is a requirement for both constitutive and regulated degradation of most cellular proteins. Moreover, these modifications also regulate many degradation-independent processes. In this series, Olsen *et al.* (3) and Lorenz *et al.* (4) review mechanistic aspects of protein modification by ubiquitin and ubiquitin-like proteins. Olsen *et al.* focus on E1 enzymes, the proteins that initiate these multistep pathways, and describe emerging structural data that reveal how E1 proteins engage their respective cognate ubiquitin-type proteins and confer specificity for this and downstream events. Lorenz *et al.* discuss the HECT class of E3 ubiquitin ligases and review the structural properties of these proteins that determine the mechanisms by which they selectively target their substrates.

## The proteasome

Fred exploited his discovery of ATP-dependent protein degradation in reticulocytes to identify the proteasome, the protease now known to catalyze the constitutive and regulated degradation of most eukaryotic intracellular proteins (5, 6). Tomko *et al.* (7) and Mao *et al.* (8) review two contemporary topics of proteasome biology: (i) the mechanisms by which cells orchestrate assembly of the multiple proteasome isoforms from their many genetically distinct subunits; and (ii) detailed mechanistic and regulatory features of the ATP-dependent 26S proteasome isoform. As described in these reviews, recent results enabled by high-resolution cryo-electron microscopy have provided important new molecular details and insights into these mechanistically complex processes.

## Prokaryotic proteasomes and proteases

The proteasome is found in all eukaryotic cells but is present in only certain prokaryotes. Fred’s early work established important roles for protein degradation in bacteria and identified and characterized a number of bacterial proteases that catalyze the process (9). Notably, several of these proteases required ATP for function and served as prototypes for features of the eukaryotic proteasome. Darwin reviews properties of the proteasome and two other ATP-dependent proteases in *Mycobacterium tuberculosis* (10). This review describes how each of these proteases plays a role in the pathogenesis of tuberculosis and how they have become attractive targets for pharmacologic control of the disease.

## Protein degradation in organelles

Although the focus of much of Fred’s mature work involved the ubiquitin-proteasome system, he had significant interests in other proteolytic systems, including those associated with lysosomes, mitochondria, and the endoplasmic reticulum. Lysosomal autophagy is a highly regulated form of protein degradation involved in protein degradation in response to many stress conditions. Rubinsztein and Puri discuss new findings about the molecular mechanisms involved in the formation of autophagosomes, the compartment that sequesters substrates for lysosomal degradation (11). Vahidi *et al.* review the molecular features and relative roles of multiple mitochondrial ATP-dependent proteases in mitochondrial function, including that of protein quality control (12). Although these endogenous proteases degrade various mitochondrial proteins, functionally defective mitochondria are degraded by a selective form of autophagy called mitophagy. This process involves ubiquitylation for subsequent processing by the lysosome, as well as action of the proteasome, an excellent example of the coordinated functioning of different proteolytic systems for a common cause. Butt *et al.* (13) review the process whereby ubiquitin is phosphorylated as part of mitophagy and describe how ubiquitin phosphorylation might be a possible biomarker for certain mitochondrial-associated diseases such as Parkinson’s disease.

## Protein degradation in muscle

Finally, Cohen *et al.* review the current field of the mechanisms and regulation of protein degradation in muscle (14). This topic is especially relevant for this series, because it was Fred’s entree into the field of protein degradation. Fred began his research career in the middle of the golden age of molecular biology, where gene expression and protein synthesis dominated thinking about cellular regulation. His earliest studies, however, revealed that changes in muscle size in response to various hormones, nutrients, and various physiologic states were determined in large part by changes in overall rates of muscle protein degradation instead of the expected changes in protein synthesis (15). Although these surprising discoveries demonstrated an important physiologic role for protein degradation, little was known at the time about the mechanisms by which these changes occurred. This gap in knowledge and the opportunities that Fred saw in this gap, set the course for the next 60 years of his work.

## Concluding perspectives

Because protein degradation is an embedded feature of so many normal cellular processes, it is not surprising that dysfunctional or abnormal protein degradation is a common feature of human diseases. Moreover, some diseases, including certain cancers, are highly dependent on protein degradation as an essential element of their pathologic phenotypes. Accordingly, pharmacologic control of protein degradation, either by corrective manipulation or by exploitation of an Achilles heel, represents an attractive strategy for drug therapy. Over 5% of the encoded human genome represents proteins involved in the ubiquitin-proteasome system and another 5% represents proteins involved in the execution and regulation of autophagy, thereby providing a rich supply of targets for this general strategy. Most of the articles in this thematic review series describe current examples or promising opportunities for treatment of various pathologies *via* the control of protein degradation. This topic is particularly relevant to the current series, because Fred played an important role in one of the best examples of this approach. In the early 1990s, Fred founded a biotech company whose efforts resulted in discovery of the first small-molecule proteasome inhibitors. These compounds were, and continue to be, important research tools for uncovering cellular roles of proteasome-dependent protein degradation. Remarkably, proteasome inhibitors were rapidly developed into drugs, such as bortezomib, that transformed the treatment of cancers, such as multiple myeloma, and have greatly extended the survival of patients who otherwise would have faced a poor prognosis (15). Proteasome inhibitors continue to be used commonly for this purpose and are in clinical trials for treatment of other cancers and diseases. Finally, recent novel therapeutic strategies such as PROTACs, molecular glues, and the many variants of these technologies, utilize the endogenous machinery and mechanisms of protein degradation for their action and hold great promise for the control of disease processes (16). At the beginning of his distinguished career, I doubt that even Fred’s fertile imagination could have anticipated the degree to which protein degradation mediates normal cellular function and how this extraordinarily rich biology offers opportunities to improve human health.
